# Supplementary figures and images for: Maternal Iron Deficiency Programs Rat Offspring Hypertension in Relation to Renin—Angiotensin System and Oxidative Stress
Source: Int J Mol Sci. 2022 Jul 27;23(15):8294. doi: 10.3390/ijms23158294 (PMC9368932; doi:10.3390/ijms23158294)

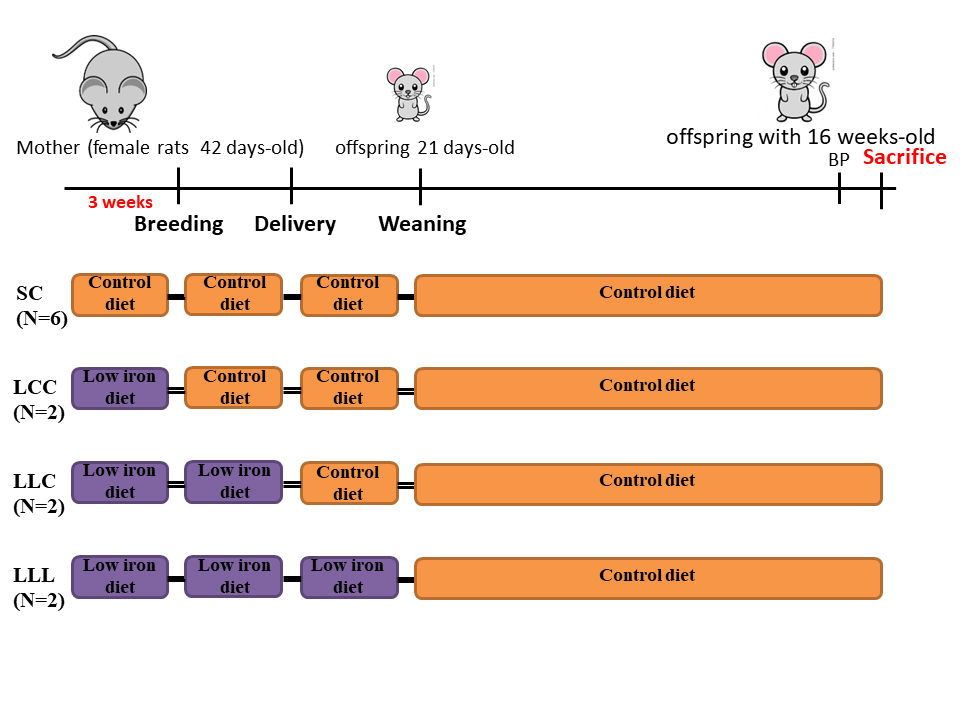

Supplement: Supplementary file 1 [file ijms-23-08294-s001.zip › ijms-1797117-Figure S1.tif]
